# Combining time-frequency and spatial information for the detection of sleep spindles

**DOI:** 10.3389/fnhum.2015.00070

**Published:** 2015-02-19

**Authors:** Christian O'Reilly, Jonathan Godbout, Julie Carrier, Jean-Marc Lina

**Affiliations:** ^1^Montreal Neurological Institute, McGill UniversityMontreal, QC, Canada; ^2^Département de Psychiatrie, Université de MontréalMontreal, QC, Canada; ^3^Dream and Nightmare Laboratory, Center for Advanced Research in Sleep Medicine, Hôpital du Sacré-CoeurMontreal, QC, Canada; ^4^Laboratoire PhysNum, École de Technologie Supérieure, Centre de Recherches MathématiquesMontreal, QC, Canada; ^5^Département de Psychologie, Université de MontréalMontreal, QC, Canada; ^6^Chronobiology Laboratory, Center for Advanced Research in Sleep Medicine, Hôpital du Sacré-CoeurMontreal, QC, Canada

**Keywords:** sleep spindles, detection, electroencephalography, time-frequency, hierarchical clustering, machine learning, pattern recognition, sleep

## Abstract

EEG sleep spindles are short (0.5–2.0 s) bursts of activity in the 11–16 Hz band occurring during non-rapid eye movement (NREM) sleep. This sporadic activity is thought to play a role in memory consolidation, brain plasticity, and protection of sleep integrity. Many automatic detectors have been proposed to assist or replace experts for sleep spindle scoring. However, these algorithms usually detect too many events making it difficult to achieve a good tradeoff between sensitivity (Se) and false detection rate (FDr). In this work, we propose a semi-automatic detector comprising a sensitivity phase based on well-established criteria followed by a specificity phase using spatial and spectral criteria. In the sensitivity phase, selected events are those which amplitude in the 10–16 Hz band and spectral ratio characteristics both reject a null hypothesis (*p* < 0.1) stating that the considered event is not a spindle. This null hypothesis is constructed from events occurring during rapid eye movement (REM) sleep epochs. In the specificity phase, a hierarchical clustering of the selected candidates is done based on events' frequency and spatial position along the anterior-posterior axis. Only events from the classes grouping most (at least 80%) spindles scored by an expert are kept. We obtain *Se* = 93.2% and *FDr* = 93.0% in the first phase and *Se* = 85.4% and *FDr* = 86.2% in the second phase. For these two phases, Matthew's correlation coefficients are respectively 0.228 and 0.324. Results suggest that spindles are defined by specific spatio-spectral properties and that automatic detection methods can be improved by considering these features.

## Introduction

EEG sleep spindles are short bursts of oscillatory activity in the 11–16 Hz frequency band during NREM sleep, especially in stage 2 sleep. This sporadic activity is a topic drawing increasingly more attention as it is thought to have an important role in the protection of sleep integrity and in the consolidation of new learning (Steriade, 2006; Dang-Vu et al., 2010; Fogel et al., 2012). Usually, the study of sleep spindles is time consuming due to the manual processing it requires. Aside from preprocessing steps such as sleep staging and artifact rejection, a polysomnographic expert has to manually identify hundreds of spindle occurrences hidden in whole-night EEG recordings, a tedious and error-prone task. Over the years, many automatic detectors have been proposed to assist or replace the experts in this task. These can be roughly split in two classes. The first one transforms the recorded signal in a new function—the *detection function*—whose amplitude is related to the probability of spindle activity. A simple threshold (or a set of thresholds) is applied to this function to decide on the presence or absence of spindle activity. This operation is typically followed by some additional criteria such as rejection of small duration events, generally <500 ms to follow standard definitions of sleep spindle (Rechtschaffen and Kales, 1968; Iber et al., 2007). Many systems following this general approach have been proposed (e.g., Schimicek et al., 1994; Huupponen et al., 2007; Devuyst et al., 2011; Babadi et al., 2012). In the second class of detectors, EEG signals are segmented in a sequence of events (i.e., epochs that are potentially associated with spindle occurrences). For each event, a set of features is extracted to better synthesize its key characteristics. Then, two approaches can be used to classify these events as spindles or non-spindles: supervised (guided by pre-annotated spindles) or unsupervised (clustering techniques finding regular subsets of events and selecting subsets that are most likely to be associated with spindle activity). Here again, many systems have been proposed in the literature (e.g., Acır and Güzeliş, 2004; Olbrich and Achermann, 2005; Ventouras et al., 2005; Sinha, 2008; Ahmed et al., 2009).

However, the detection of an important proportion of false positives is a persistent problem observed with these automated detectors when compared to expert scoring. This issue has often been hidden by reports of apparently highly specific systems which large numbers of false positives were masked by the important asymmetry between spindle vs. non-spindle events (O'Reilly and Nielsen, 2013; O'Reilly and Nielsen, in revision). Looking at the false detection rate (instead of specificity) reveals this important weakness. In this context, achieving a satisfactory tradeoff between sensitivity (Se) and false detection rate (FDr) proved to be challenging.

In this work, we propose a two-step detector which aims to decrease the FDr by combining a *sensitivity phase* based on well-established criteria to a *specificity phase* using spatial and time-frequency criteria. This approach mixes both types of classification approaches previously described. In the *sensitivity phase*, putative events are first detected from the wavelet representation of the EEG recordings and then selected as those with large sigma index—a measure proposed by Huupponen et al. (2007) as a ratio of specific spectral bands—and high amplitude in the spindle frequency band. The threshold used in this selection process is based on the rejection of a null hypothesis (*p* < 0.1) stating that the considered event is not a spindle. The non-parametric model of the null hypothesis is constructed from events occurring in spindle-free epochs, e.g., in REM stage. In the *specificity phase*, hierarchical clustering of detected events is performed using the spectral and the topographical (anterior vs. posterior localization) properties of spindles. This spatio-spectral classification is motivated by evidences of a dichotomy in sleep spindles: one class occurs in frontal regions and has lower frequencies; another class is characterized by higher frequencies and a more centro-parietal topography (Werth et al., 1997; Zeitlhofer et al., 1997; Anderer et al., 2001; De Gennaro and Ferrara, 2003; Martin et al., 2013). Then, classes grouping a large proportion of events scored as spindles by an expert are selected. In this phase, the detector tries to reject as many false positives as possible—hence effectively biasing the detection threshold toward specificity—without rejecting too many true positives. Interestingly, parameters for such clustering can be learned from a small sample of expert detections and then be generalized automatically to the whole night.

## Materials and methods

### Preprocessing

#### Signal mixture

A first preprocessing step consists in locally averaging the EEG signals to obtain one highly informative signal out of the *N_c_* EEG channels available. This is made possible by the fact that the spindle activity is generally relatively synchronous across the scalp, with maximal apparition delays between sensors generally below 25 ms (O'Reilly and Nielsen, 2014b). We consider the following virtual channel:

(1) $$s^{\left(m\right)}=m^{T}S$$

where *m* is a vector of *N_c_* components specifying weights associated with every channel of this mixture. This vector is normalized with a L_1_ norm (i.e., elements sum to unity) and defines what we call a *montage*. The *S* matrix has a dimension *N_c_* × *N_t_* and is obtained by simply stacking together the signals from the *N_c_* channels, each one containing *N_t_* time samples.

#### Time-frequency representation

**Figure 1:**
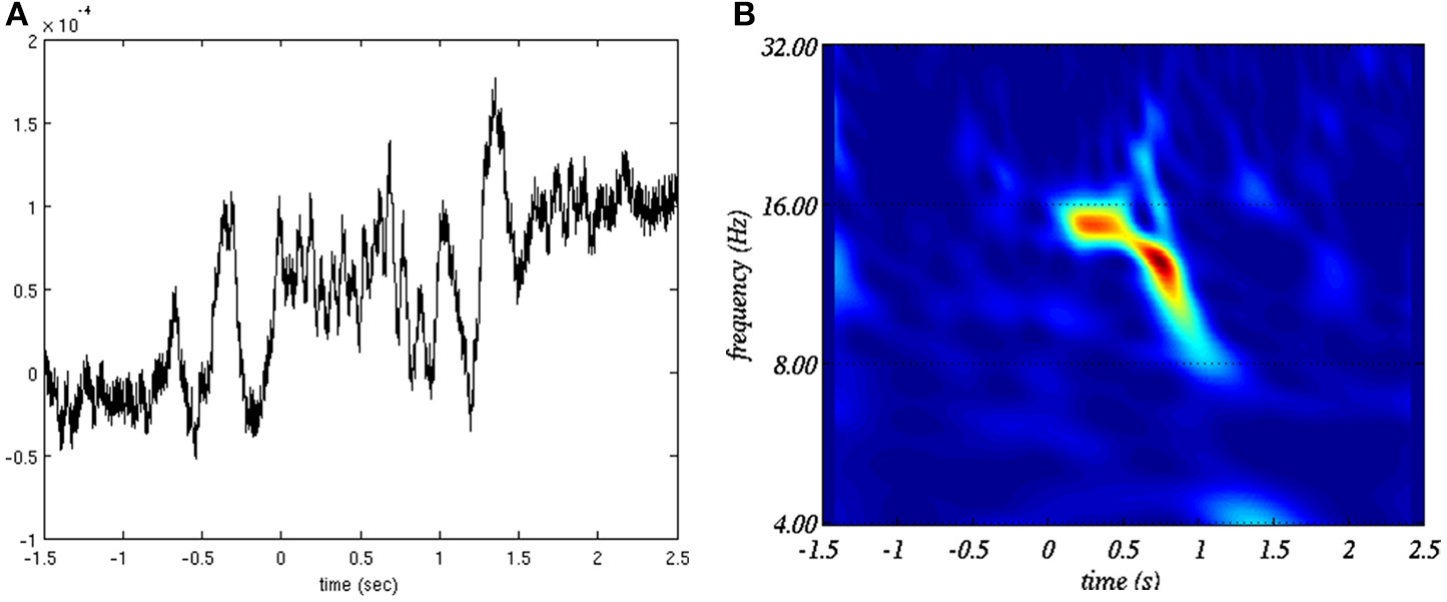
**Example of sleep spindle (A) in time domain and (B) in the time-frequency plane**.

Spindle activity was assessed in the time-frequency plane using the Continuous Wavelet Transform (CWT). This transform is defined as follows:

(2) $$W(a,b)=\frac{1}{2\pi }\int_{-\infty }^{+\infty }\Psi_{\beta ,\gamma }^{*}\left(a\omega \right)S\left(\omega \right)e^{i\omega b}d\omega$$

with *a* and *b* being parameters associated respectively with scale (i.e., inverse of frequency) and time. $S(\omega )=\int_{-\infty }^{+\infty }s(t)e^{-i\omega t}dt$ is the Fourier transform of the signal *s*^(*m*)^(*t*),^*^ indicates the complex conjugate, and Ψ_β,γ_ (ω) is a wavelet in the frequency domain. For this study, we used the Morse wavelet (Lilly and Olhede, 2009, 2010):

(3) $$\Psi_{\beta ,\gamma }\left(\omega \right)=H\left(\omega \right)c_{\beta ,\gamma }\omega^{\beta }e^{-\omega^{\gamma }}$$

with *c*_β,γ_ being an irrelevant normalization factor and *H*(ω) being the Heaviside function (null everywhere but for ω ≥ 0 where it is equal to 1). We set γ = 20 and β = 10. These values were found to provide the best tradeoff between time and frequency resolution for sleep spindle representation. See Figure 1 for an example of time-frequency representation of a sleep spindle using this transform.

#### Wavelet ridge and temporal markers in the time-frequency plane

Computing (2) produces a matrix *W*^(*m*)^ of CWT coefficients *w*^(*m*)^_*i*, *j*_ at time *t_j_* and frequency *f_i_* = *f*_0_/*a_i_*, *f*_0_ being the main frequency of the wavelet Ψ_β,γ_ (ω). For each time sample, we considered the local maximal amplitude along frequencies of the spindle spectral band. We then computed the time course of those wavelet maxima, i.e.,:

(4) $$d\left(t_{j}\right)=\underset{1\leq i\leq N_{f}}{\max}\left|w_{i,j}^{\left(m\right)}\right|$$

Named *ridge* (Delprat et al., 1992), this piecewise continuous path across the time-frequency map *W*^(*m*)^ quantifies the power of instantaneous frequency in the signal. To be sensitive to the spindle frequency band, it was computed using frequencies sampled from 10 to 16 Hz with 0.1 Hz resolution, resulting in *N_f_* = 61 frequencies per time sample.

To allow for a parsimonious assessment of spindle features, the ridge was first marked according to the local maxima of the *d*(*t_j_*) function:

(5) $$t^{max}=\left\{t_{j}\in t:\dot{d}\left(t_{j}\right)\dot{d}\left(t_{j+1}\right)<0\;and\;\dot{d}\left(t_{j}\right)>0\;\right\}$$

with *ḋ* being the time derivative of d. These maxima are considered as time markers for the putative events (i.e., one event is counted for each item in the *t^max^* set) in the time-frequency plane.

#### Feature computation for the sensitivity phase

Two features are computed for signal detection in the *sensitivity phase*. The first one is the ridge amplitude at the maxima: $\tilde{x}$*^amp^_n_* = *d*(*t^max^_n_*), with *n* = 1, 2,…, *N* and *N* being the number of elements in the *t^max^* set. The second feature is a spectral sigma ratio similar to what was proposed by Huupponen et al. (2007) but computed using the modulus of the activity in the time-frequency space (|*W*^(*m*)^|) in the 4–40 Hz range:

(6) $$\begin{array}{l} {\tilde{x}}_{n}^{sigma}=\frac{2a_{\sigma }}{a_{\alpha }+a_{\beta }}\\ =\frac{2max\left\{{\left|W\right|}_{\left[10.5-16\text{Hz};\;t_{n}^{max}\right]}\right\}}{mean\left\{{\left|W\right|}_{\left[4-10\text{Hz};\;t_{n}^{max}\right]}\right\}+mean\left\{{\left|W\right|}_{\left[20-40\text{Hz};\;t_{n}^{max}\right]}\right\}}\; \end{array}$$

**Figure 2:**
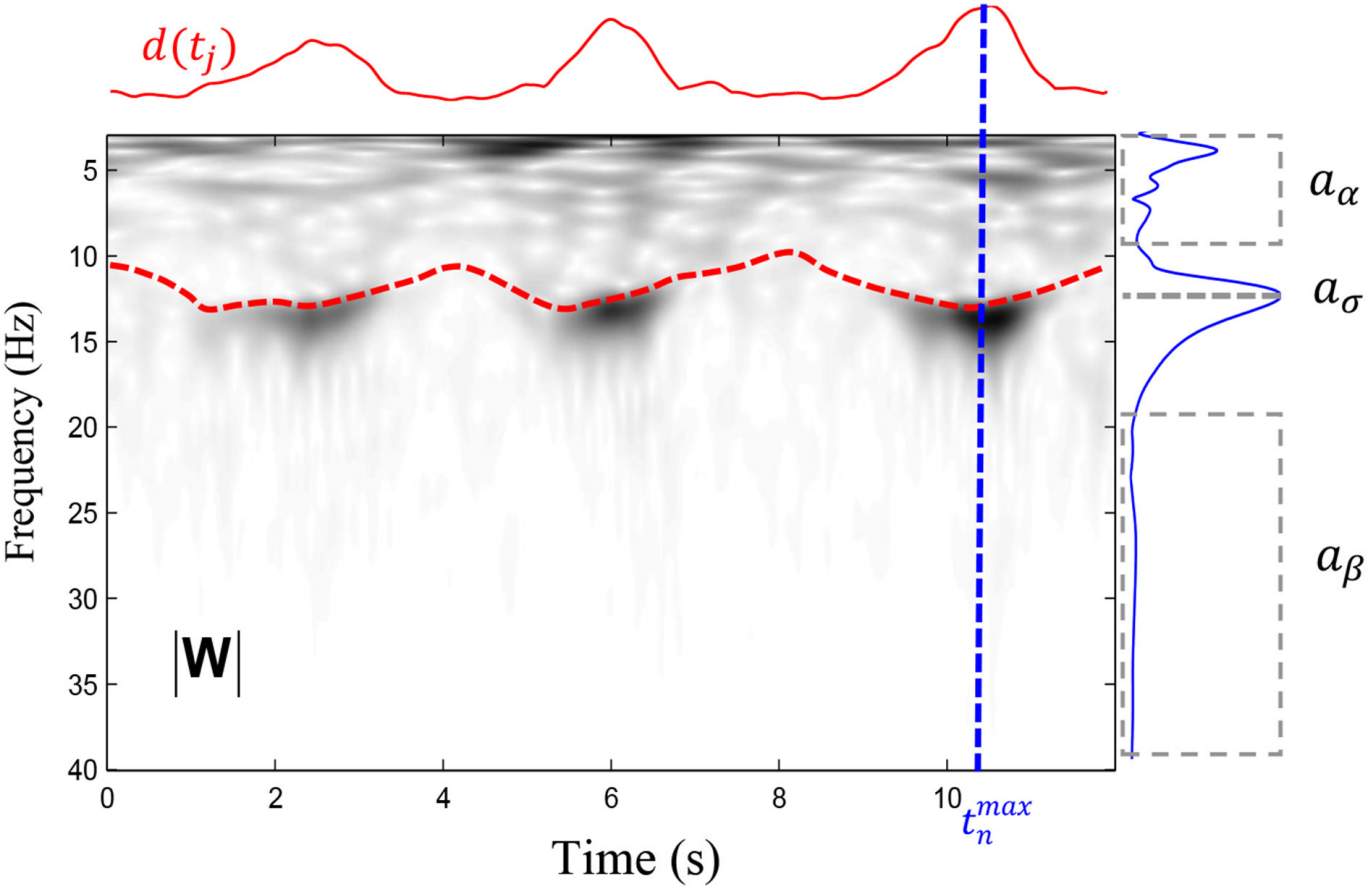
**Illustation of the variables entering in the computation of $\tilde{x}$*^sigma^_n_* as defined in (6)**. The time-frequency plane (|*W*|), in gray levels, is represented by the modulus of wavelet coefficients computed for frequencies between 4 and 40 Hz with 0.1 Hz resolution. The red dashed line shows the ridge computed in the 10–16 Hz band whereas the solid red line shows the *d*(*t_j_*) function defined in (4). A maximum has been detected at time *t^max^_n_* and the variation of the coefficients (i.e., the instantaneous spectrum) at the time *t^max^_n_* is shown by the solid blue line. From this spectrum, we extract the average amplitude in two intervals (shown by dashed boxes comprising respectively the low and the high frequencies) to obtain $a_{\alpha }=mean\left\{{\left|W\right|}_{\left[4-10\;Hz;\;t_{n}^{max}\right]}\right\}$ and $a_{\beta }=mean\left\{{\left|W\right|}_{\left[20-40\;Hz;\;t_{n}^{max}\right]}\right\}$. We further take the maximal amplitude in the spindle band to obtain $a_{\sigma }=max\left\{{\left|W\right|}_{\left[10.5-16\;Hz;\;t_{n}^{max}\right]}\right\}$.

This index increases with narrow band activity having a peak in the 10.5 − 16 Hz band. Compared to the root-mean-square amplitude of the activity in the sigma band—a measure often used for spindle detection (e.g., Schimicek et al., 1994; Molle et al., 2002; Clemens et al., 2005; Schabus et al., 2007; Warby et al., 2014)—it has the advantage of penalizing muscular artifacts (20 − 40 Hz) and signs of arousal (4 − 10 Hz). It might, however, be adversely impacted by the increase of theta and beta activity associated with sleep spindles (Vyazovskiy et al., 2004). This measure was chosen since it represents a state-of-the-art approach for spindle detection and it has shown to perform reasonably well in previous studies (Huupponen et al., 2007, 2008; Sheng-Fu et al., 2012; O'Reilly and Nielsen, in revision). An example of corresponding values is shown in Figure 2.

#### Feature computation for the specificity phase

**Figure 3:**
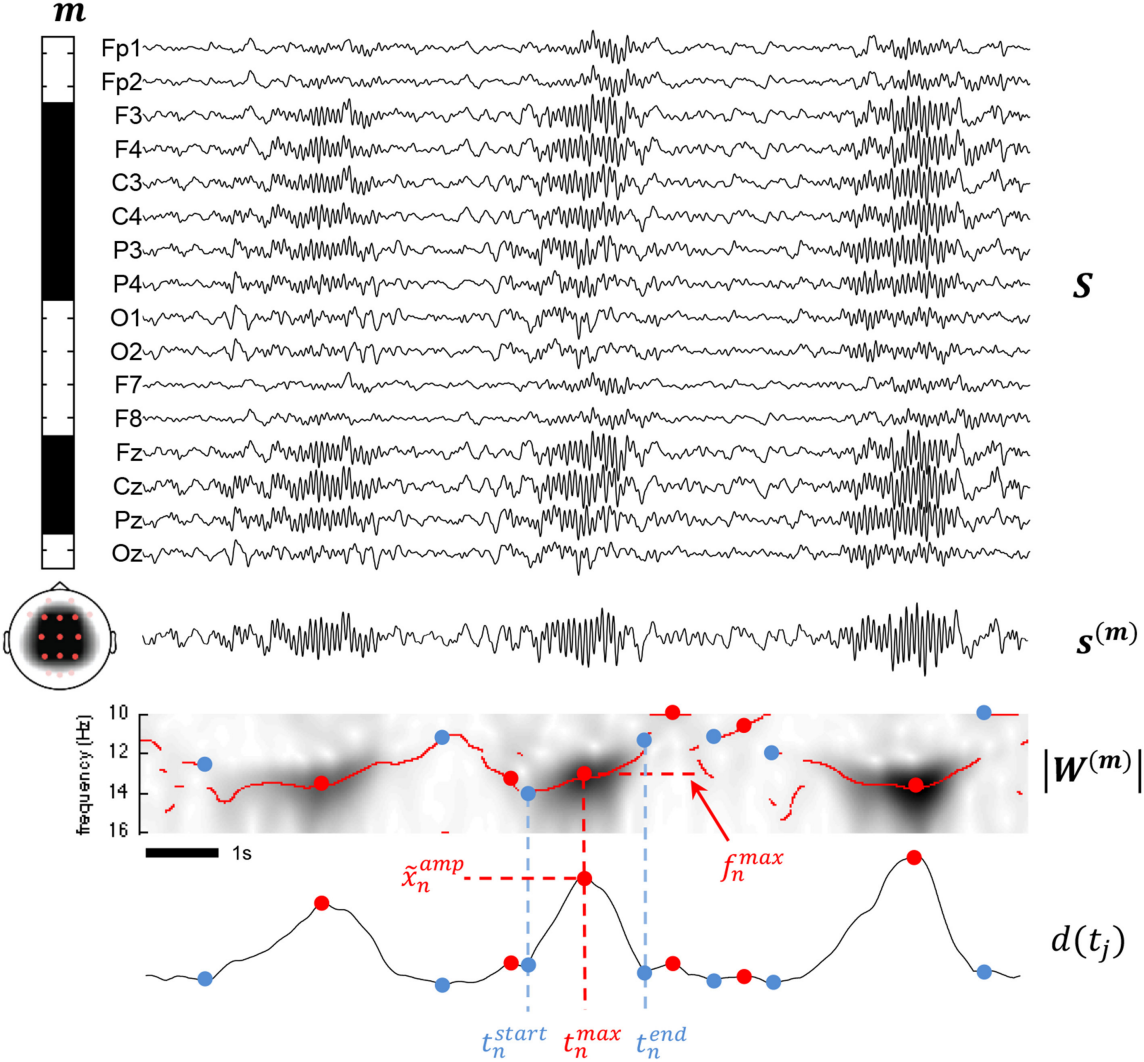
**This figure summarizes various aspects of the proposed methodology**. The montage defined by the *m* vector is illustrated by the leftward band displaying binary weights including frontal, parietal, and central channels and excluding the other channels from the mixture. Below the band, a scalp map shows the topological coverage of this montage (red dots for included electrodes, pink for excluded). At the right of the montage band, all available channels are stack in a matrix S. The mixture signal *s*^(*m*)^ is obtained by matrix multiplication of *m* and *S* as described in (1). Applying the CWT to *s*^(*m*)^ and taking the modulus, we obtain a time-frequency map |*W*^(*m*)^ as shown. Using (4) on |*W*^(*m*)^, we compute the *d*(*t_j_*) detection function used to determine the times *t^start^*, *t^max^*, and *t^end^*. $\tilde{x}$*^amp^^n^* is obtained as *d*(*t^max^_n_*) and *f^max^_n_* as *f_i_* with $i=\underset{\begin{array}{c} 1\leq i\leq N_{f}\\ t_{j}=t_{n}^{max} \end{array}}{\text{argmax}}\;\left|w_{i,j}^{\left(m\right)}\right|$.

Two other features defined between 0 and 1 are computed in the *specificity phase*. The first feature assesses the main frequency mode of a putative spindle *n*:

(7) $${\tilde{x}}_{n}^{freq}={\frac{f_{n}^{max}-f_{1}}{f_{N_{f}}-f_{1}}|}_{f_{1}=10,\;f_{N_{f}}=16}$$

where *f^max^_n_* = *f_i_* with $i=\underset{1\leq i\leq N_{f}}{\text{argmax}}\left|w_{i,j}^{\left(m\right)}\right|$ and *j* is such that *t_j_* = *t^max^_n_*. Figure 3 summarizes important concepts introduced so far.

The second feature captures the location of spindle activity along the anteroposterior axis of the scalp. To compute this value, we consider the first principal component (PC) of a 500 ms window centered around *t^max^_n_*. This spatial eigenvector represents a normalized topography over the channels, and its components correspond to the relative weight for each channel. Being the first PC, this topography picks the larger variability of the multivariate signal over the analyzed window. Then, the position of the channel with maximal weight can be considered representative of the scalp localization of the event centered around *t^max^_n_*. Channel positions are specified as (*x_n_*, *y_n_*) coordinates in the 10-5 system (Oostenveld and Praamstra, 2001) mapped to a flat top view of the scalp as specified in the EEG1005.lay montage file of the FieldTrip software (Oostenveld et al., 2011). Only the *y_n_* value is used for spindle detection given the observation of different types of spindles in relation with their anteroposterior position (Dehghani et al., 2011; Martin et al., 2013; O'Reilly and Nielsen, 2014b). The feature for localization along the medial axis is defined as:

(8) $${\tilde{x}}_{n}^{med}=y_{n}+0.5$$

such that it is normalized to the [0, 1] range.

#### Threshold computation

Two extra quantities are used to set thresholds needed by the algorithm. Both are derived from information related to the timing and space location of the spindles given *a priori* by a gold standard, typically an expert. The first one is a sleep stage related feature, $\tilde{x}$*^stage^_n_*, which value is an integer between 0 and 5 (0: awake; 1: NREM1; 2:NREM2, 3:NREM3; 4:NREM4; 5:REM). This value is defined on the current sleep stage at the moment of *t^max^_n_*. The second feature indicates whether an event occurred during a time window associated with a spindle also visually identified by an expert on channels Fz, Cz, or Pz. That is, $\tilde{x}$*^expert^_n_* = 1 if *t^max^_n_* is co-occurring with a spindle labeled on any of these three channels. Otherwise, a zero value is attributed.

It is worth highlighting that the proposed detection technique rests on “point” features (i.e., features evaluated at a given point in time) and not on features computed on time windows. Thus, the detector set instantaneous markers for sleep spindles without explicit duration.

### Sensitivity phase

The goal of this phase is to detect as many true spindles as possible, missing only a small proportion, at the cost of a relatively high amount of false positives. In this *sensitivity phase*, we test the null-hypothesis stating that ${\tilde{x}}_{n}^{sensitive}=\left[\begin{array}{cc} {\tilde{x}}_{n}^{amp} & {\tilde{x}}_{n}^{sigma} \end{array}\right]$ is not associated with a spindle. For this assessment, a sample of the null−hypothesis, i.e., non−spindle events, is built from $\tilde{x}$*^sensitive^_n_*of all events with $\tilde{x}$*^stage^_n_* = 5. Although, it has been proposed that isolated spindles can occur in REM (Rechtschaffen and Kales, 1968), this is controversial. In the same line of thought, sleep spindles could also be present in transition pages marked as REM but containing some proportion of NREM sleep. Nevertheless, presence of spindles in pages marked as REM should be rare and should therefore have little impact on our statistics.

Decision thresholds are computed separately for both features. This implicitly postulate statistical independence, a reasonable hypothesis given the relatively low correlation reported (about 0.25 according to Huupponen et al., 2007) between these two features. Two thresholds—τ^*amp*^ and τ^*sigma*^—are obtained as the value of $\tilde{x}$^*amp*^ and $\tilde{x}$^*sigma*^ at the (1 − α) percentile of the distribution of the non-spindle events. That is, we compute thresholds that should fail to reject at most a proportion α of false positives. As discussed in O'Reilly and Nielsen (2014a), such an approach sets the expected false detection rate (FDr; complete definition in Table 1, Section Performance Assessment) to:

(9) $$FDr=\frac{\alpha \kappa }{P_{\%}}$$

**Table 1:** **Definition of performance metrics used in this paper**.

**Meaure**	**Formula**
Sensitivity (Se)	$S_{e}=\frac{N_{TP}}{N_{FN}+N_{TP}}$
Specificity (Sp)	$S_{p}=\frac{N_{TN}}{N_{FP}+N_{TN}}$
False positive rate (FPr)	$FP_{r}=\frac{N_{FP}}{N_{FP}+N_{TN}}=1-S_{p}$
False Discovery Rate (FDr)	$FD_{r}=\frac{N_{FP}}{N_{FP}+N_{TP}}$
False positive proportion (FPp)	$FP_{p}=\frac{N_{FP}}{N_{FN}+N_{TP}}$
Matthew's correlation coefficient (MCC)	$\text{MCC=}\frac{{\text{N}}_{TP}*{\text{N}}_{TN}-{\text{N}}_{FP}*{\text{N}}_{FN}}{\sqrt{\begin{array}{l} \left({\text{N}}_{TP}+{\text{N}}_{FP}\right)\left({\text{N}}_{TP}+{\text{N}}_{FN}\right)\\ \;\;\;\;\;\;\;\;\;\left({\text{N}}_{TP}+{\text{N}}_{FP}\right)\left({\text{N}}_{TP}+{\text{N}}_{FN}\right) \end{array}}}$
F1 score	$F_{1}=\frac{2{\text{N}}_{TP}}{2{\text{N}}_{TP}+N_{FP}+N_{FN}}$
Cohen κ	$\begin{array}{l} \;\kappa =\frac{\text{X-}\left({\text{N}}_{TN}+{\text{N}}_{TP}\right)N_{e}}{X-N_{e}{}^{2}}\text{with}\\ X=\left({\text{N}}_{TP}+{\text{N}}_{FP}\right)\left({\text{N}}_{TP}+{\text{N}}_{FN}\right)\\ \;+\left({\text{N}}_{FP}+{\text{N}}_{TN}\right)\left({\text{N}}_{FN}+{\text{N}}_{TN}\right) \end{array}$

with κ being the proportion of false positives in the tested sample and P_%_ the proportion of the tested sample not rejected by this threshold. Although we cannot compute the value for the FDr because we lack an estimate for κ, we can obtain an upper bound $\tilde{\text{FDr}}$ using:

(10) $$\tilde{FDr}=\frac{\alpha }{P_{\%}}$$

With these thresholds, we can now define a subset ***X*** of selected candidates as:

(11) $$X=\left\{X_{m}\right\}=\left\{{\tilde{x}}_{n}\in \tilde{X}:{\tilde{x}}^{amp}\geq \tau^{amp}\;and\;{\tilde{x}}^{sigma}\geq \tau^{sigma}\right\}$$

### Specificity phase

**Figure 4:**
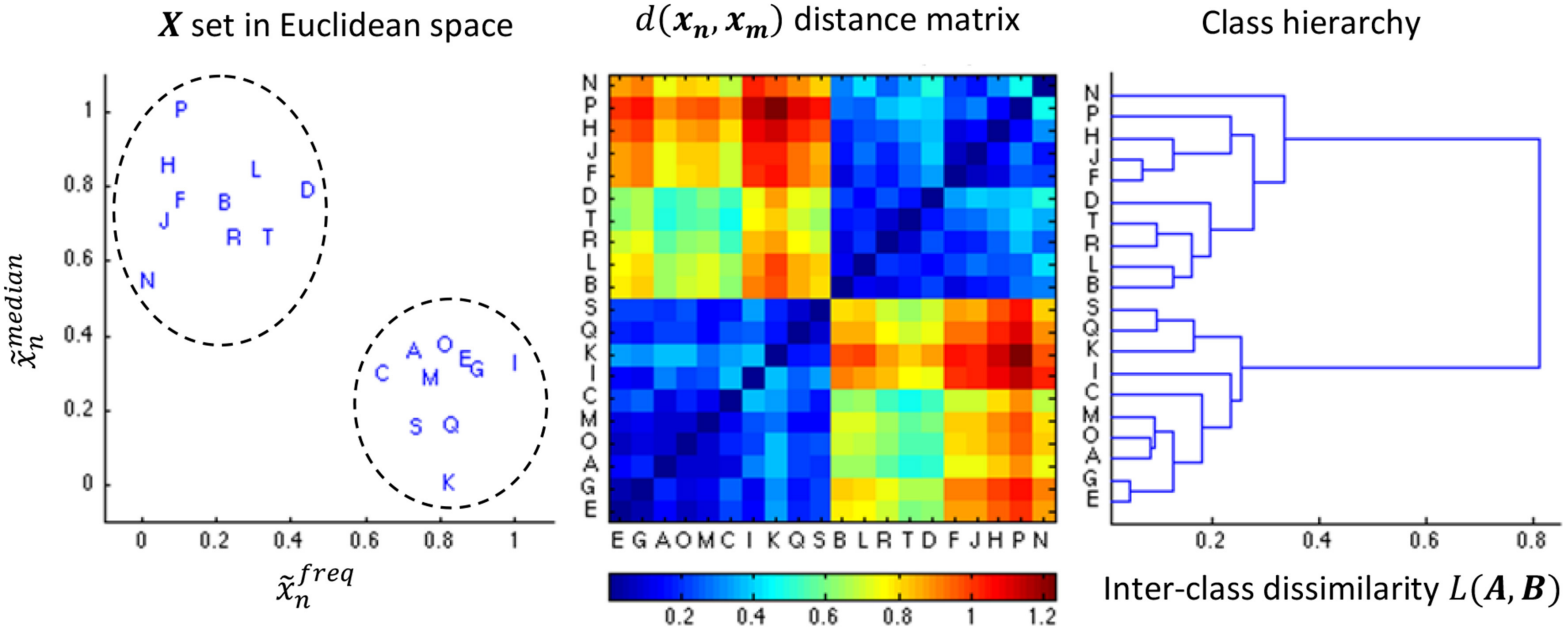
**Illustration of the AHC algorithm**. An example of 20 events characterized by the medial position and the frequency (both normalized to the unit range) is shown in the leftward pane. The middle pane shows the color coded distance matrix corresponding to these 20 events. Finally, the right most pane shows the resulting dendrogram. The dendrogram is sequentially split into more classes in a top-down fashion, stopping the decomposition as soon as we reach two classes since (in this specific example) both contains 10 samples such that $\frac{\left|B\right|}{\left|A\right|}=\frac{10}{10}\geq 0.6=r$.

Previous selection of events is used as input to the *specificity phase* which tries to keep only selected candidates corresponding with spindles, as identified by an expert. A partition of selected candidates in homogenous classes of events is performed using the *ascending hierarchical classification* (AHC) algorithm (Timm, 2002). This technique starts with every item of *X* being considered as a singleton class and iteratively regroups together the two most similar classes until only one class regrouping all items is left. The outcome of such a process can be represented as tree graph called a *dendrogram* (see Figure 4 for an example). The AHC algorithm is defined by a *metric* and a *linkage criterion*. The former defines how we assess the distance between two items whereas the latter do the same for two classes of items. In our case, we used the Euclidean distance as metric:

(12) $$d\left(x_{n},x_{m}\right)=\sqrt{\sum_{i}{\left(x_{n}^{\left(i\right)}-x_{m}^{\left(i\right)}\right)}^{2}}$$

where the *i* index iterates over elements of *x_n_* and *x_m_* vectors. For linkage criterion, we used the average distance *d*(*x_n_*, *x_m_*) between items of two classes *A*, *B* ∈ *X* defined as:

(13) $$L\left(A,B\right)=\frac{1}{\left|A\right|\left|B\right|}\sum_{x_{a}\in \;A}\sum_{x_{b}\in \;B}d\left(x_{a},x_{b}\right)$$

with |*A*| and |*B*| standing for the cardinality of classes *A* and *B*, respectively. Figure 4 illustrates the use of the AHC algorithm.

The final clustering is obtained by cutting the dendrogram at the maximal value of inter-class dissimilarity subject to the inequality:

(14) $$\frac{\left|B\right|}{\left|A\right|}\geq r$$

with *A* and *B* being respectively the largest and second largest classes. This criterion tends to favor homogeneity of class sizes. A value *r* = 0.6 was chosen in this study because it was found to be a good tradeoff between accepting only equally sized classes (i.e., *r* = 1.0) and allowing much disparate classes such as one big cluster associated with a very small outlier class (i.e., *r* → 0.0). Classes obtained that way are then sorted in descending order according to their number of *expert events* (i.e., events scored as spindles by the expert). For the *specific detection*, only events belonging to the first *N_class_* classes are labeled as spindles, with *N_class_* being the smallest number of classes grouping at least 80% of the expert events.

### Performance assessment

For assessing performances, we used a terminology borrowed from confusion matrices. Four classification outcomes can be encountered in the dual-class problem considered here: true positives (TP), false positives (FP), true negatives (TN), and false negatives (FN). If we consider a variable *x^selected^_n_* which takes 1 when the nth event is designated as a spindle by the algorithm and otherwise takes 0, these four cases are obtained as follow:

(15) $$TP\Leftrightarrow {\tilde{x}}_{n}^{selected}=1\;\wedge \;{\tilde{x}}_{n}^{expert}=1$$

(16) $$TN\Leftrightarrow {\tilde{x}}_{n}^{selected}=0\;\wedge \;{\tilde{x}}_{n}^{expert}=0$$

(17) $$FP\Leftrightarrow {\tilde{x}}_{n}^{selected}=1\;\wedge \;{\tilde{x}}_{n}^{expert}=0$$

(18) $$FN\Leftrightarrow {\tilde{x}}_{n}^{selected}=0\;\wedge \;{\tilde{x}}_{n}^{expert}=1$$

Counts of each outcome are labeled respectively *N_TP_*, *N_TN_*, *N_FP_*, and *N_FN_* and are the constitutive elements of the metrics used to score our algorithm (see Table 1). Here, we are measuring agreement using a “by-event” approach (Warby et al., 2014) where an agreement is marked if and only if a specific point (i.e., the local maximum of the ridge) is within one of the spindle windows scored by the expert. The total number of events N_e_ (i.e., *N_e_* = *N_TP_* + *N_FP_* + *N_FN_* + *N_TN_*) is defined by the segmentation described in section Wavelet ridge and temporal markers in the time-frequency plane.

### Implementation

The detector has been implemented as a “process” in Brainstorm (Tadel et al., 2011). The source code is available from the corresponding author.

### Sample

We tested our algorithm on polysomnograms recorded in a hospital-based sleep laboratory from 9 (7 women, 2 men) young (mean ± standard deviation: 22.6 ± 2.4 years old) and healthy subjects. Recording was performed at 256 Hz using a Vita-port-3 System (low-passed at 70 Hz with 1-s time constant) and the data were recorded using the Columbus software from TEMEC Instruments (Kerkrade, Netherlands). We used a standard 10–20 EEG sensor grid (C3, C4, Cz, F3, F4, Fz, F7, F8, O1, O2, Oz, P3, P4, Pz, T3, T4, T5, T6, Fp1, Fp2) with a 10 kΩ ear-linked reference as well as bipolar chin EMG, ECG, and EOG. Sleep stages were scored by a certified polysomnographer with 15 years of experience according to modified rules of Rechtschaffen and Kales (1968) adapted for 20-s epochs. Muscle artifacts were automatically detected (Brunner et al., 1996) and visually confirmed. Sleep spindles were scored by the same expert on Fz, Cz, and Pz channels in NREM sleep epochs. Spindle scoring was performed on raw signals according to the rules of the AASM (Iber et al., 2007). Sleep stage distribution per subject (Table S1) as well as number of spindles scored per derivation per subject (Table S2) are provided as Supplementary Documents.

Every recording was sanctioned by the ethics review board of the Hôpital du Sacré-Coeur de Montréal and participants gave informed consent.

## Results

### Sensitive detection

#### Montage selection

We tested six different montages to study their effect on the sensitive detection: m_1_ corresponds to frontal channels Fp1, Fp2, F7, and F8; m_2_ to occipital channels O1, O2, and Oz; m_3_ to channels F3, F4, C3, C4, P3, P4, Fz, Cz, and Pz; m_4_, m_5_, and m_6_ to only Fz, Cz, and Pz, respectively. To avoid biasing toward some of these selected channels, we used equal weights for every channel of the montages (i.e., weights equal to 1/N_i_ where N_i_ equals the number of channels included in the montage).

**Figure 5:**
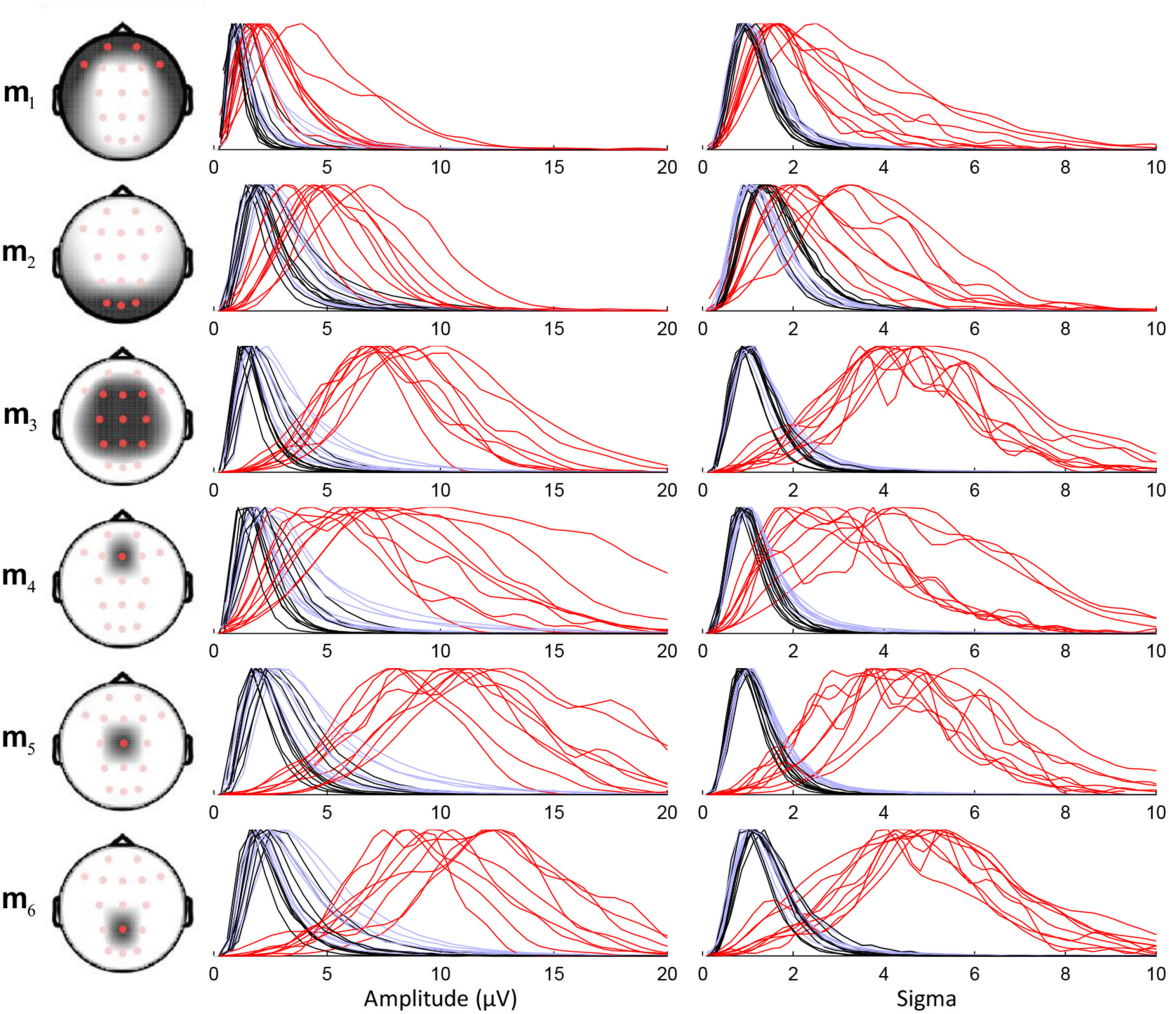
**Density plots with normalized maximal amplitude for the distribution of sensitive detection features (amplitude and sigma index) for six different montages**. The head drawings show the topographical coverage of each montage. Feature distributions are plotted as three separate lines per subject: red for detected spindles corresponding with expert scoring, black for events scored as spindles by the algorithm in REM epochs only, and light blue for detected events during REM and NREM sleep.

Performance of the sensitive detection depends on the capacity of the chosen montage to discriminate between the sleep spindles (in red in Figure 5) and the non-spindle events (in black). For example, the small overlap between these two sets of curves in *m*_3_ indicates a good discriminative power. We note that some simpler montages (e.g., montages *m*_5_ and *m*_6_ using only Cz and Pz, respectively) also show similarly good performances. Lower discrimination is obtained using only Fz (*m*_4_) or using in general only frontal and prefrontal (*m*_1_) or occipital (*m*_2_) scalp channels. Results presented subsequently are obtained using *m*_3_.

#### Performance evaluation

**Figure 6:**
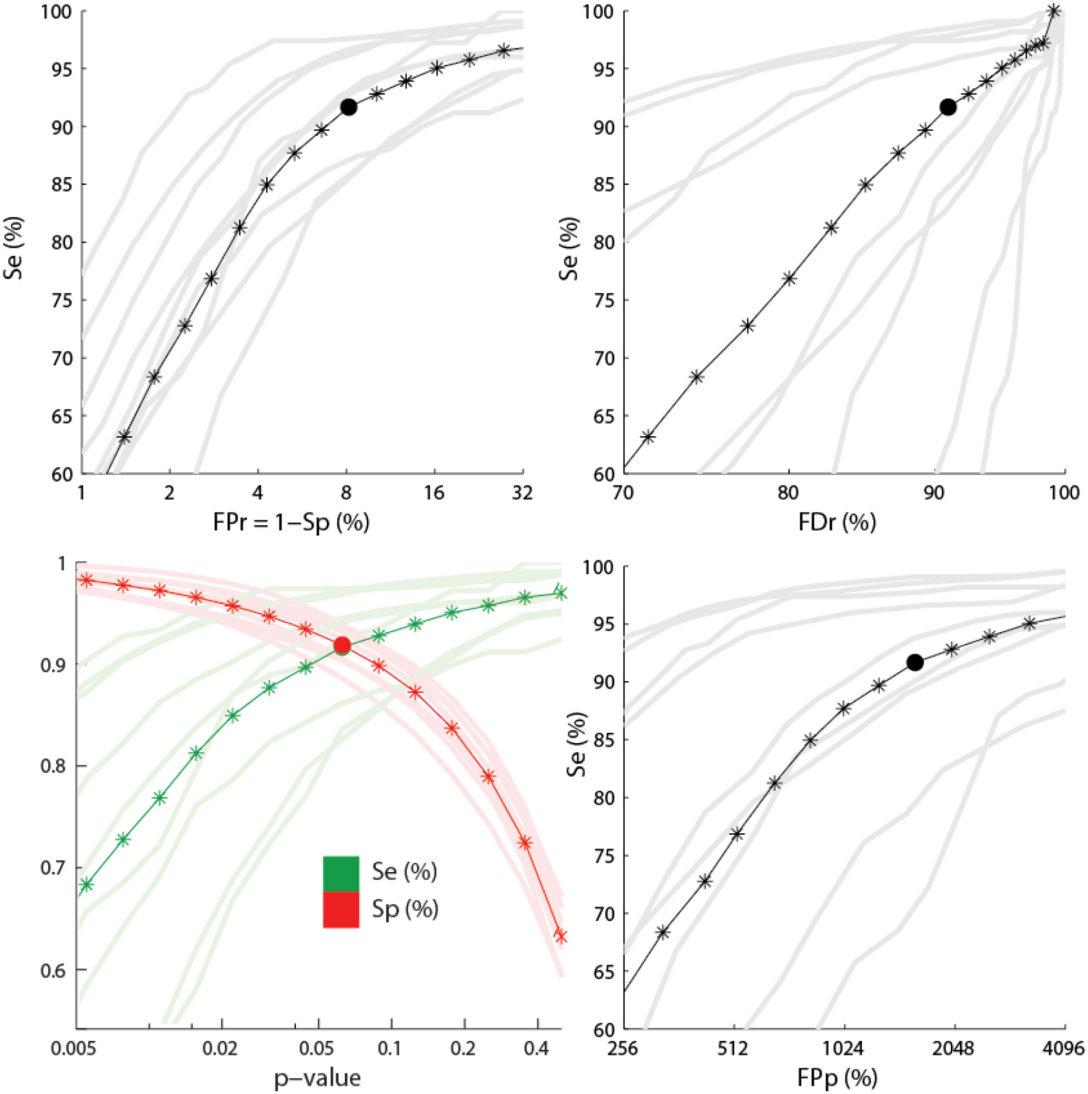
**Performance for the sensitive detection**. The lower left panel shows the sensitivity (green) and the specificity (red) as function of the *p*-value used for feature thresholding. The three other panels show ROC-like curves using FPr, FDr, and FPp. Dark colors show average statistics whereas light colors correspond to performances for every subject. Stars indicate the positions where the statistics have been computed. Segments between stars are obtained by linear interpolation. Filled circles show points where Se = Sp. The x-axes are plotted using a logarithmic scale.

Results from a receiver operating characteristic (ROC) curve analysis are presented in Figure 6.

Averages and standard deviations (SD) of the performance statistics are reported in Table 2 for the conditions *S_e_* = *S_p_* and α = 0.1, with the second condition focusing slightly more on sensitivity. One should note that this table do not report specificity since this statistic has little value in evaluating spindle detectors because it systematically takes high values given the small proportion of positive to negative cases (i.e., spindle vs. non-spindle) (O'Reilly and Nielsen, 2013). For the same reason, the reader should be cautious in interpreting the ROC curves in Figure 6 since only the portion with large specificity is meaningful. Lower specificity are associated with prohibitively high FDr, something not visible in ROC curve (O'Reilly and Nielsen, 2013).

**Table 2:** **Average ± SD value for performance statistics when *S_e_* = *S_p_* and when α = 0.1 for the sensitive and the specific phase**.

**Mesure**	**Sensitive phase**		**Specific phase**
	***S_e_* = *S_p_***	**α = 0.1**	**α = 0.1**
*Se*	92.1 ± 3.0%	93.2 ± 4.8%	85.4 ± 7.4%
*FPr*	7.9 ± 2.7%	11.0 ± 2.1%	4.5 ± 1.7%
*FDr*	89.1 ± 8.3%	93.0 ± 4.2%	86.2 ± 6.1%
*FPp*	1779.6 ± 1936.6%	2177.1 ± 2110.3%	730.3 ± 598.9%
*MCC*	0.28 ± 0.13	0.23 ± 0.08	0.32 ± 0.08
*F1*	0.19 ± 0.13	0.13 ± 0.07	0.23 ± 0.09
κ	0.17 ± 0.13	0.11 ± 0.06	0.22 ± 0.09

### Specific detection

**Figure 7:**
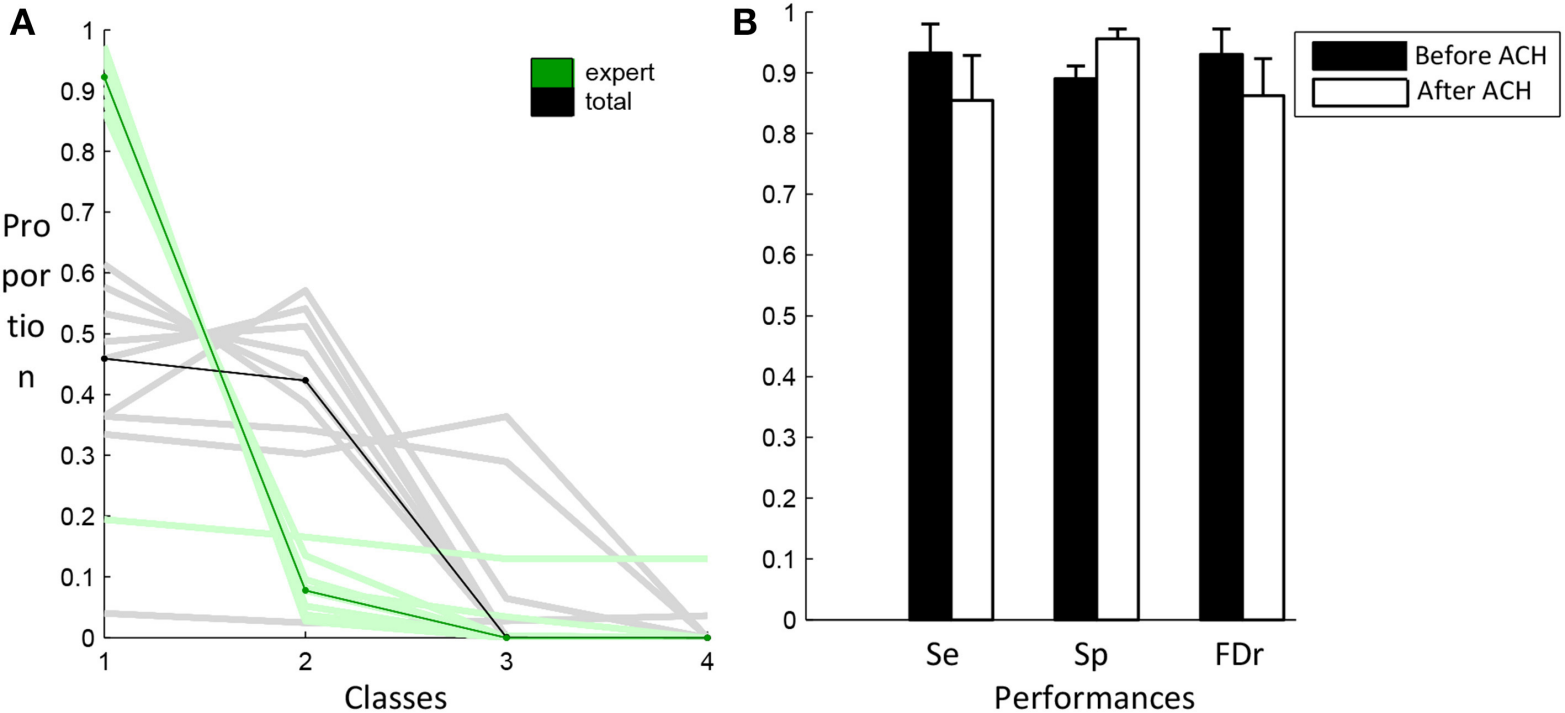
**Results from the AHC algorithm**. In **(A)**, the distribution of expert (green) and total (black) events in the four first classes. Light color lines correspond to individual subjects whereas dark lines correspond to the median values across subjects. The graph in **(B)** compares the performance of the algorithm after the sensitivity phase (black) and after the specificity phase (white).

Figure 7A shows the proportion of spindles scored by the expert (green) and the proportion of total events (black) contained in the four classes produced by the clustering algorithm. These classes are sorted in decreasing number of expert events. Lines of lighter color are used for individual subjects while darker lines are used for the median across subjects. As specified in the Materials and Methods section, events selected by the specificity phase are those belonging to the first classes regrouping at least 80% of the expert events. As can be seen, only one class is required to reach this criterion. Except for S4, using only one class, we can keep more than 80% of the expert events while keeping about only 50% of the total number of events initially selected in the previous sensitivity phase. In Figure 7B, classification performances obtained with this criterion (white bars) are compared to the performance obtained before the application of this criterion (black bars).

**Figure 8:**
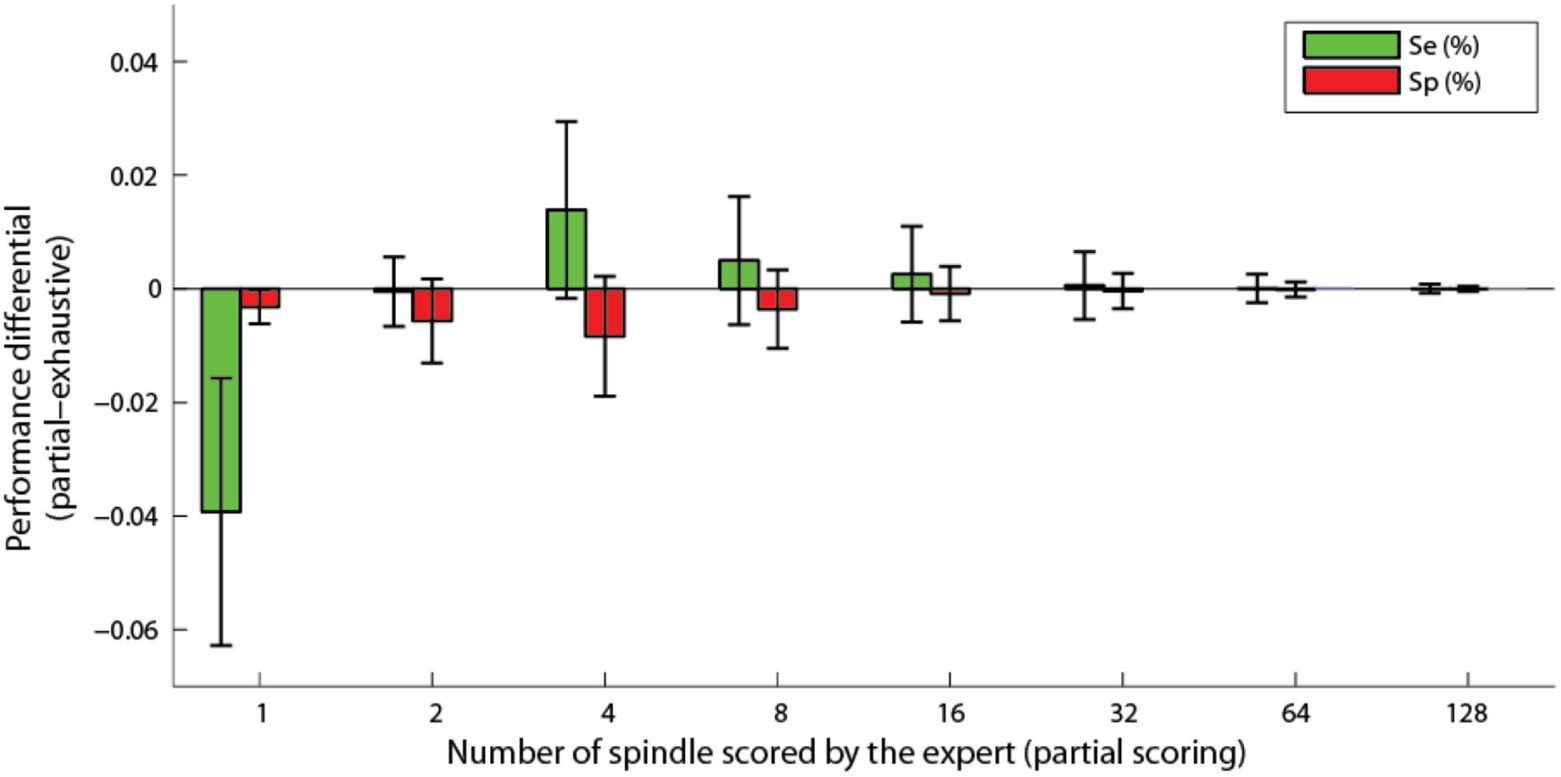
**Differential of sensitivity and specificity for partial scoring compared to exhaustive scoring, as a function of the number of spindles scored by an expert**. Bars show the average difference and whiskers the standard deviation.

It should be noted that results of Figure 7 are obtained using all available expert scoring. This is in average 390 spindles per subject. We also tested whether the proposed algorithm could be used with a reduced number of sleep spindles sampled by the expert. Hence, bootstrapping over 500 repetitions has been performed using randomly selected subsets of 1, 2, 4, 8, 16, 32, 64, and 128 scored events. Figure 8 shows the differential (partial minus exhaustive scoring) in sensitivity and specificity. Subject S4 was excluded from this analysis because the unusual clustering in four equal size classes for this subject produced unstable results when using small subsets of expert scorings. As can be seen, the performances are not significantly degraded by partial scoring using about 16 or 32 spindles visually scored by an expert.

### Characteristics of detected spindles

This section compares automatically detected spindles with those identified by the expert.

#### Frequency and medial position

**Figure 9:**
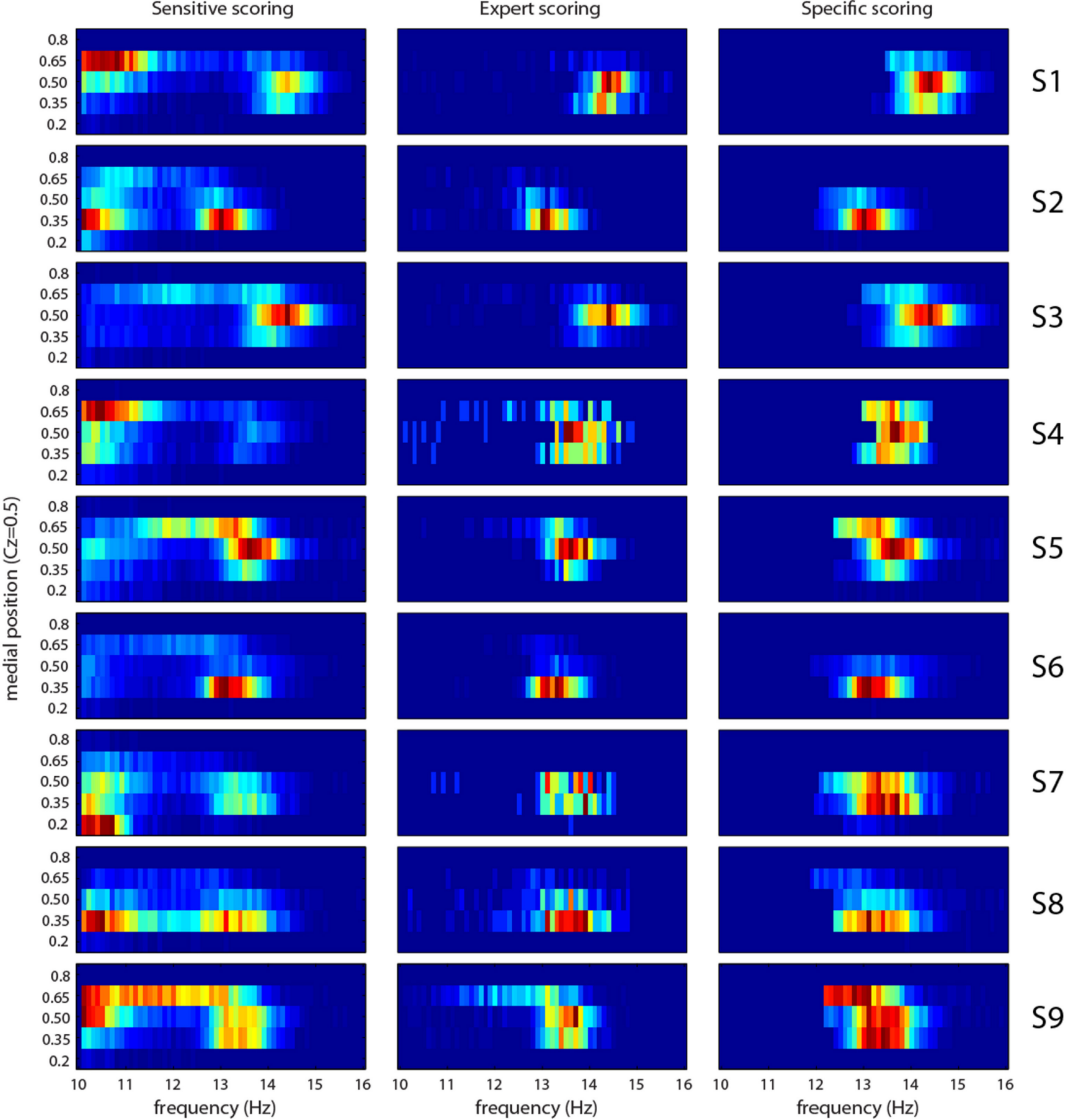
**Joint distribution of frequency and medial position (0.15 is occipital and 0.9 is prefrontal) features for each subject (S1–S9 rows) and for sensitive detection (first column), expert scoring (second column), and specific detection (third column)**. Cold colors (toward blue) represent small amplitude, hot colors (toward red) large amplitudes.

Figure 9 shows the joint distribution of $\tilde{x}$*^freq^_n_* and $\tilde{x}$*^med^_n_*. The later value varies between 0.15 (occipital) and 0.9 (pre-frontal). In general, distributions of features after the sensitivity phase suggest two classes of events, although the frontier separating these classes is blurry and varies from subject to subject. From the literature, we would expect a fast (higher frequency) centro-parietal (0.35 < $\tilde{x}$*^med^_n_* < 0.5) class and a slow (lower frequency) frontal ($\tilde{x}$*^med^_n_* > 0.5) class. This behavior is observed for subjects S1, S3, S4, and S9, and to a lesser extent for S5 and S6. In S2 and S8, we do observe fast and slow classes, but both in centro-parietal region. In S7, the slow class is located in occipital region ($\tilde{x}$*^med^_n_* = 0.2) suggesting alpha rhythm contamination. Actually, most spindles scored by the expert tend to be in the fast centro-parietal class. Spindles automatically scored after the specificity phase follow this trend (comparison of results in second and third column of Figure 9).

#### Average spindle

**Figure 10:**
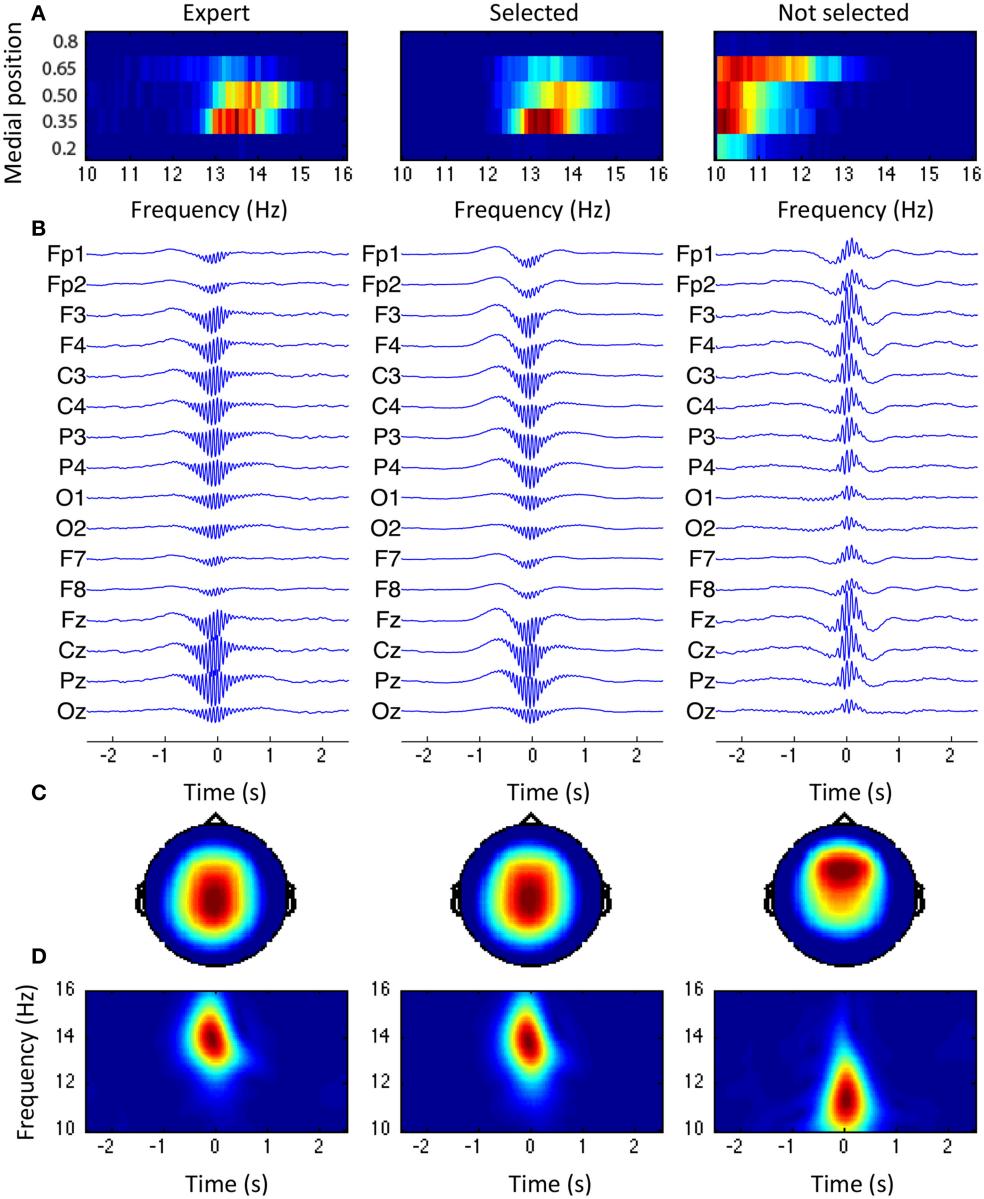
**Grand average across subjects**. From left to right: spindle scored by the expert, events from the selected class, and events from the rejected classes. **(A)** Joint distribution of the frequency and the medial position. **(B)** Average spindle. **(C)** Topography of the first principal component obtained through PCA. **(D)** Time-frequency plane for the average spindle, using the montage specified by the topography shown in **(C)**.

Figure 10 shows the grand average for spindles scored by the expert, spindles selected by the sensitive detection, and events accepted during sensitivity phase but rejected by the specificity phase. In Figure 10A, the joint distribution for the frequency and the medial position is shown. In Figure 10B, the average signal for each channel is shown using a 5-s window centered around *t^max^_n_*. Averages are first computed within subjects and then between subjects. At each level, signals are time-aligned by maximizing the cross-correlation of the central 500 ms of activity in the 10–16 Hz band. Figure 10C shows the first principal component (i.e., the component with the highest variability) computed on the central 500 ms window of the between-subjects averaged signal (band-passed in the 10–16 Hz band with a 5th order Butterworth filter). Finally, Figure 10D shows the time-frequency plot computed using the CWT (Morse wavelets with γ = 20 and β = 20) of the between-subjects average signal using the montage specified by the topographic vector of the first principal component (i.e., as shown in panel C).

Topographies in panel C and joint distributions in panel A both tend to support the existence of two classes of events with fast (13–14 Hz) centro-parietal activity and a slow (10–12 Hz) more diffuse activity generally located in more frontal areas. The expert visually scored mainly the first class and so did our specific selection. Spindles are shown to be in phase with a ~1 Hz component, reproducing the observations about slow wave/spindle phase-amplitude coupling previously reported (Molle et al., 2002; Kokkinos and Kostopoulos, 2011).

#### Spindles across sleep cycles

**Figure 11:**
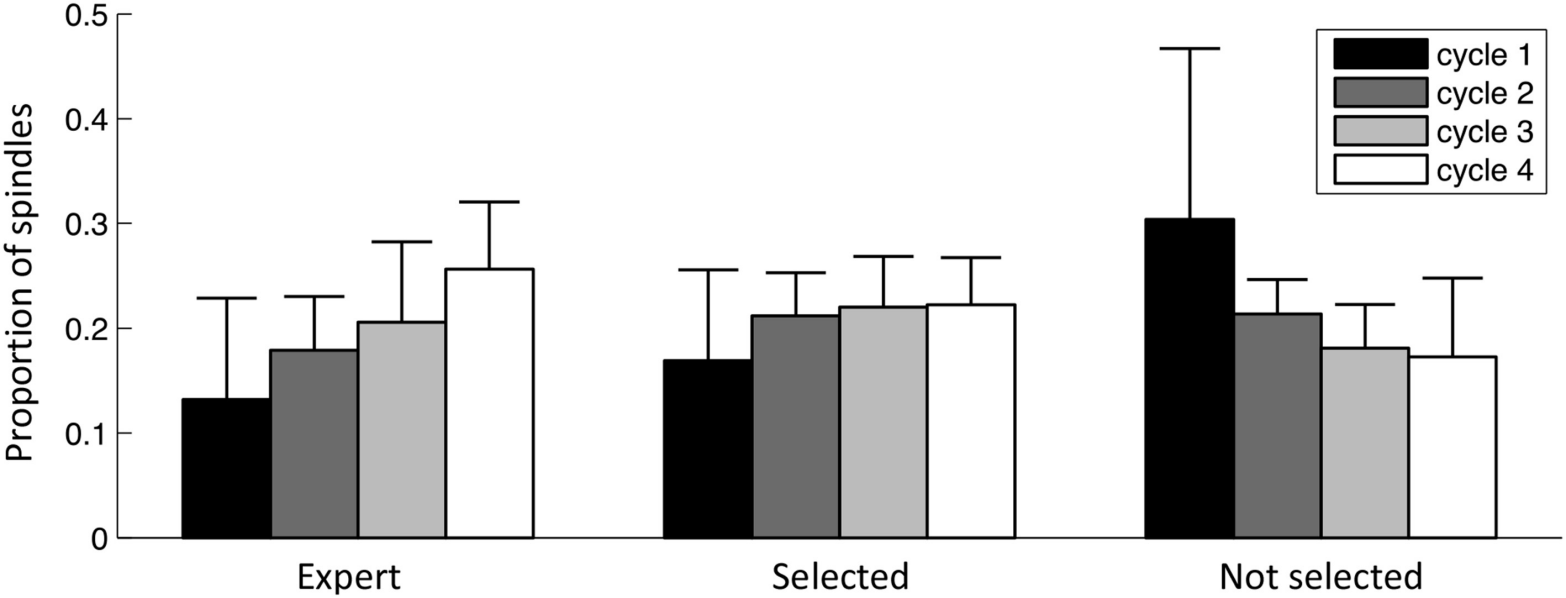
**Spindle distribution across sleep cycles**. Bars indicate the mean value across subjects, whiskers indicate standard deviations. Values are normalized for subjects and classes (i.e., each of these three sets of bars sums to the unity).

Figure 11 shows how the proportion of spindles in each of the fours first sleep cycles evolves for 1) events selected by the expert, 2) events selected by the specific detection, 3) events rejected by the specific detection. Sleep cycles were defined according to Aeschbach and Borbely (1993): one cycle is a sequence of a NREM period followed by a REM period. The NREM period starts at the first epoch of NREM sleep and terminates at the first REM epoch. The REM period terminates only if the next 15 min are free of REM epochs. At least four cycles were present in every subject. As can be seen, in both expert scoring and detector expert class, spindles show a similar trend with an increasing density from the beginning to the end of the night. The non-expert class shows an inverse tendency.

## Discussion

The goal of this study was to tackle the problem of high false detection rates in sleep spindle scoring. The strategy adopted was to split the problem in two steps, a sensitive detection (unsupervised) and a specific detection (supervised). In the following sections, we discuss various aspects of our method and results.

### Detection montage

The approach described in section Montage selection provides the possibility of compressing a multivariate signal (coming from different channels) into a univariate signal using a specific montage. In this study, based on standard definition of spindles (Rechtschaffen and Kales, 1968; Iber et al., 2007) and on the current knowledge on spindle topography, we favored a montage weighting equally frontal (F3, Fz et Fz), central (C3, Cz, C4) and parietal (P3, Pz, P4) channels and excluding the others. This montage failed to show a clear superiority compared to montages using single channels (e.g., Cz or Pz). One should note, however, that our gold standard (i.e., expert scoring) assessed spindles only on Fz, Cz, and Pz, a fact that could have contributed in favoring montage using only these channels. Also, further work is needed to confirm whether an improved detection can be achieved by tailoring more accurately the montage. Nonetheless, the approach has interesting applications for future developments as it provides a great flexibility to apply arbitrary montage to EEG signals, as shown for computation of Figure 10D.

### Adaptive segmentation and time-frequency representation

In our method, we proposed an adaptive segmentation that split the whole night in a sequence of contiguous events. This segmentation was performed using the ridge of the continuous wavelet transform of the time series for the chosen montage. For simplicity, and because it provides a good tradeoff between temporal and spectral resolution, the Morse wavelet was used. Its parameters (γ = 20 and β = 10) were chosen using visual inspection. One should note, however, that β is the most sensitive parameter. Large values tend to over-smooth and reduce the temporal resolution whereas too small values tend to under-smooth resulting in appearance of amplitude modulation of the time-frequency plane at higher frequencies (closer to the spindle band). Higher values for β might be adequate in more noisy environments—such as for EEG signals collected during functional magnetic resonance imaging (fMRI)—to shift the tradeoff between temporal resolution and noise rejection.

### Characteristics of selected spindles

Most spindles scored by the expert were rapid (>13 Hz) and in the centro-parietal region of the scalp. The usual slow/fast dichotomy was not observed (see Figure 9). This result could be attributed to a specific detection bias of this expert and needs to be corroborated by looking at scorings from other expert. Notably, however, this slow-fast dichotomy has mostly been reported in studies using automated spindle detections. Since experts score spindles with enough amplitude to be visually discriminated from background activity, part of the false positives could also be false negatives from experts. Also, in *post-hoc* investigations, we noted that spindles detected in Fz are simultaneously detected in the fast centro-parietal class, which tends to indicate that spindles detected in Fz are observations of the same phenomenon producing faster and stronger spindles in Cz and Pz.

The coupling observed here and elsewhere (Molle et al., 2002, 2011) between the phase of a slow ~1 Hz oscillation and the amplitude in the spindle band (see Figure 10B) warrants further investigation on spindle relationship with other frequency bands. Aside from slow waves, spindles have also been reported to be coupled with gamma (30–100 Hz) oscillations (Ayoub et al., 2012). This kind of features might be useful in increasing specificity of future detectors.

Spindle distribution across the first four sleep cycles behaved similarly for the class of spindles selected by the specific detection and the expert detection (fast spindles occurring in more posterior locations) and is shown to increase progressively across the night. This profile agrees with the evolution of the sigma band (12–14.75 Hz) reported by De Gennaro and Ferrara (2003). Events not selected at the specificity phase are generally slower with more anterior localization and have an inverse tendency: their density decreases across night. De Gennaro and Ferrara (2003) have reported that the power in the delta band (0.5–4.75 Hz) shows a similar trend, motivating the investigation of whether events in these classes are coupled with the activity in this lower frequency band. Also, since spindles have been detected on all NREM states, a more thorough analysis would be necessary to disambiguate the role of sleep stages in this trend.

### Dependence on expert scoring

The proposed system is semi-automatic, requiring an expert for stage scoring and partial spindle annotation. Stage scoring is a standard operation generally performed before manual or automatic spindle detection. However, if one does not want to score whole nights, only some spindle-free epochs (such as REM epochs) can be scored manually and fed to the algorithm. Alternatively, automatic sleep scoring algorithms can be used (Anderer et al., 2005). Although these algorithms do not perform as well as experts, they should be reasonably accurate to discriminate some classes of spindle-free epochs (wake, REM) vs. epochs possibly containing spindles (NREM stages).

As for partial spindle scoring, our results suggest that only 20 spindles per subject are sufficient to benefit from the supervised classification. Thus, the expert scoring burden is relatively small with this detector. Of course, as for any supervised system, the scoring will be as biased as the expert. Thus, using expert consensus (Warby et al., 2014) on small number of spindles instead of single-expert scoring is worth more investigation. Another future avenue is to automate the clustering using some *a priori* knowledge instead of expert scoring. To implement this, we could for example take advantage of the fact that events detected by the sensitivity phase naturally tend to show two classes plus some outliers. Using the k-mean clustering algorithm with *k* = 2 to extract the centroid of the two classes and reject outliers that are not close enough to these centers is likely to give interesting results.

### Gold standard in spindle scoring

It should be noted that the performance assessment reported in this study is limited by the relatively low reproducibility of our gold standard: expert scoring. With relatively low inter-rater agreement between expert scorers (around 86% in Campbell et al., 1980); 61 ± 6% and Cohen κ of 0.52 ± 0.07 (Wendt et al., 2014); around 0.2 and 0.4 Cohen κ in DREAMS and MASS open-access databases, respectively (O'Reilly and Nielsen, in revision), development of automated detectors will stay rather limited until the subjective assessment of spindle by experts is transcended and supplanted by a more robust, objective, and commonly agreed upon gold standard (O'Reilly and Nielsen, in revision).

### Clustering

The clustering algorithm has shown to be able to dichotomize sleep spindles in the fast/slow classes reported in the literature for all but one subject. Topography of spindles is not always stable across time and the clustering might be sensible to this inhomogeneity. The properties of the clustering process will require more investigation on larger samples to better understand when it fails, what it indicates, and how it can be corrected. Also, although both fast and slow classes are generally correctly identified, the slow class was rejected by our automated system because our expert ignored tentative spindles from this class. Whether this behavior is typical in expert scoring is still to be evaluated. Similarly, whether some variables (e.g., the expert) impact on the minimal number of scored spindles needed to obtain a reliable clustering is still an open question. In our investigation, only a small number of spindle per subject (about 20) were shown to be sufficient.

Furthermore, given the somewhat low inter-rater agreement between experts reported in literature (Wendt et al., 2014), using an expert consensus measure could present great advantages (Warby et al., 2014). One should note, however, that such a strategy would probably bias scoring toward classes with high amplitude and high signal-to-noise ratio.

## Conclusion

The principal contribution of this paper is to propose a two-step methodology to address first the sensitivity and second the specificity of spindle detection. For this last step, we proposed an unsupervised clustering using spectral and positional (along the medial axis) features to take into account the fast-posterior/slow-anterior spindle dichotomy followed by a supervised class selection. Some other original contributions proposed in this paper are: (1) the compression of channel arrays into a univariate signal using a fixed montage, (2) using the ridge of a time-frequency map to segment the signal and transform it into a detection function, and (3) using *p*-values for setting selection thresholds, based on a null-hypothesis elaborated from the spindle-free periods during sleep (e.g., REM).

Acceptable classification results have been obtained with *Se* = 85.4%, *FDr* = 86.2%, and *MCC* = 0.32. Although these results are similar to those available in literature, a more thorough comparison is not reported here since such an analysis would be unreliable due to the large confounding impact of using different expert scorings. For example, MCC has been shown to vary between 0.25 and 0.55 for a same detector depending on the database and the expert scoring (O'Reilly and Nielsen, in revision). Also, the assessment methodology would not be completely comparable because of the use of a particular segmentation paradigm impacting on the counts of positive/negative events. A more thorough assessment performed with comparison against other standard detection algorithms on an open-access database (e.g., O'Reilly et al., 2014) is warranted. Such an assessment is however outside of the scope of the present paper and is a topic for future investigations.

Nevertheless, it appears that there is room for improvement since the obtained agreement is below what is expected from experts. The proposed system might be enhanced by adding specific features that are known from literature to be associated with sleep spindles such as circadian and homeostatic influences (Knoblauch et al., 2003), phase coupling with slow oscillations (Molle et al., 2002), age (Martin et al., 2013), and so on. A thorough analysis of whether adding such features can indeed improve spindle detection would however be necessary since correlations between spindles and these other variables are emerging when averaging over a large number of events. Thus, they might prove not to be specific enough to improve detection of single events and can even have a detrimental impact on automatic detection, as formalized by the No Free Lunch theorem (Wolpert and Macready, 1997).

### Conflict of interest statement

The authors declare that the research was conducted in the absence of any commercial or financial relationships that could be construed as a potential conflict of interest.
