# Supplementary material for: Combining time-frequency and spatial information for the detection of sleep spindles
Source: Front Hum Neurosci. 2015 Feb 19;9:70. doi: 10.3389/fnhum.2015.00070 (PMC4333813; doi:10.3389/fnhum.2015.00070)
Supplement: Supplementary file 1 [file Table1.PDF]

Combining time-frequency and spatial information for the detection of sleep spindles

Christian O'Reilly, Jonathan Godbout, Julie Carrier et Jean-Marc Lina

Supplementary documents

Table S1. Number of minutes and percentage of time spent in each sleep stage.

Subject	Wake minutes (%)	NREM1 minutes (%)	NREM2 minutes (%)	NREM3 minutes (%)	NREM4 minutes (%)	REM minutes (%)	TOTAL minutes (%)
**S1**	19.0 (3.73 %)	19.7 (3.86 %)	259.7 (50.95 %)	55.0 (10.79 %)	21.7 (4.25 %)	133.3 (26.16 %)	508.3 (99.74 %)
**S2**	42.0 (8.55 %)	30.3 (6.17 %)	247.0 (50.27 %)	37.7 (7.67 %)	3.3 (0.68 %)	119.7 (24.36 %)	480.0 (97.69 %)
**S3**	19.3 (3.79 %)	10.0 (1.96 %)	286.3 (56.18 %)	49.3 (9.68 %)	11.0 (2.16 %)	132.0 (25.90 %)	508.0 (99.67 %)
**S4**	48.3 (8.95 %)	20.3 (3.76 %)	305.0 (56.45 %)	52.7 (9.75 %)	10.7 (1.97 %)	72.7 (13.45 %)	509.7 (94.32 %)
**S5**	104.7 (19.42 %)	66.0 (12.24 %)	272.3 (50.53 %)	5.7 (1.05 %)	0.0 (0.00 %)	90.0 (16.70 %)	538.7 (99.94 %)
**S6**	16.7 (3.41 %)	23.7 (4.85 %)	324.0 (66.35 %)	1.3 (0.27 %)	0.0 (0.00 %)	109.3 (22.39 %)	475.0 (97.27 %)
**S7**	88.7 (16.53 %)	37.3 (6.96 %)	272.0 (50.71 %)	41.3 (7.71 %)	2.3 (0.44 %)	67.3 (12.55 %)	509.0 (94.90 %)
**S8**	12.0 (2.86 %)	12.3 (2.94 %)	263.3 (62.75 %)	40.0 (9.53 %)	5.0 (1.19 %)	86.7 (20.65 %)	419.3 (99.92 %)
**S9**	7.0 (1.54 %)	13.0 (2.87 %)	237.3 (52.35 %)	52.7 (11.62 %)	7.0 (1.54 %)	135.7 (29.93 %)	452.7 (99.85 %)
**Mean**	**39.7 (7.64 %)**	**25.9 (5.07 %)**	**274.1 (55.17 %)**	**37.3 (7.56 %)**	**6.8 (1.36 %)**	**105.2 (21.34 %)**	**489.0 (98.15 %)**
**Standard deviation**	**35.2 (6.41 %)**	**17.5 (3.13 %)**	**27.4 (5.88 %)**	**20.2 (4.12 %)**	**6.9 (1.34 %)**	**26.8 (6.01 %)**	**36.2 (2.24 %)**

Table S2. Number of spindles scored by the expert.

Subject	Fz	Cz	Pz	Union[1]	Distinct[2]
**S1**	99	223	283	605	391
**S2**	44	110	212	366	258
**S3**	344	625	401	1370	752
**S4**	23	45	64	132	97
**S5**	117	206	180	503	308
**S6**	96	375	702	1173	775
**S7**	8	50	49	107	75
**S8**	40	109	190	339	256
**S9**	268	360	403	1031	602
**Total**	**1039**	**2103**	**2484**	**5626**	**3514**
**Mean**	**115**	**234**	**276**	**625**	**390**
**Standard deviation**	**116**	**190**	**203**	**460**	**263**

[1] Union: sum of the events detected in Fz, Cz, and Pz.

[2] Distinct: without duplicates, a duplicate corresponding to a spindle scored at same time on more than one channel.
